# Update on Acute Disseminated Encephalomyelitis in Children and Adolescents

**DOI:** 10.3390/children8040280

**Published:** 2021-04-06

**Authors:** Serena Massa, Adriana Fracchiolla, Cosimo Neglia, Alberto Argentiero, Susanna Esposito

**Affiliations:** Pediatric Clinic, Pietro Barilla Children’s Hospital, Department of Medicine and Surgery, University of Parma, Via Gramsci 14, 43126 Parma, Italy; serena.massa92@gmail.com (S.M.); adriana.fracchiolla@gmail.com (A.F.); negliamino@gmail.com (C.N.); aargentiero85@gmail.com (A.A.)

**Keywords:** acute disseminated encephalomyelitis, ADEM, central nervous system, children, demyelinating disease

## Abstract

Acute disseminated encephalomyelitis (ADEM) is an immune-mediated, inflammatory demyelinating disease of the central nervous system (CNS) that usually affects children and young adults after an infection or vaccination. The presence of several conditions mimicking ADEM, added to the lack of specific biomarkers, makes diagnosis potentially hard. Prompt diagnosis is necessary to start adequate treatment to improve the clinical course and long-term outcome. Because of its heterogeneity in both clinical presentation and course, challenges remain in establishing the most appropriate therapeutic approach in each patient. The aim of this review is to provide an update on management of this disease with a focus on acute treatment and to give suggestions for future research. We showed that there are currently no guidelines that help clinicians manage ADEM and therapeutic decisions are often made on a case-by-case basis. Further studies are necessary to identify clinical, laboratory, and instrumental criteria that could be correlated with outcomes and guide clinicians in choosing when and what treatment should be given in each case.

## 1. Introduction

Acute disseminated encephalomyelitis (ADEM) is an immune-mediated, inflammatory demyelinating disease of the central nervous system (CNS) that usually affects children and young adults after an infection or vaccination [1]. The presence of several conditions mimicking ADEM, added to the lack of specific biomarkers, makes diagnosis potentially hard. Prompt diagnosis is necessary to start adequate treatment to improve the clinical course and long-term outcome [2]. Because of its heterogeneity in both clinical presentation and course, in addition to the absence of outcome predictive factors, the therapeutic approach differs from case-to-case. Currently, case reports, small observational studies, and expert opinions are the only basis for therapy [2]. Indeed, the low incidence of this disease and its frequent spontaneous recovery make it difficult to conduct large-sized, randomized controlled studies. Challenges remain in establishing the most appropriate therapeutic approach in each patient. 

Actually, ADEM treatment is based on nonspecific immunotherapy, in accordance with the supposed pathogenesis of the syndrome itself and the analogy with multiple sclerosis (MS) [2]. Indeed, although the exact pathogenesis is still not completely known, the most accurate hypothesis is about immune-mediated damage to the CNS. The aim of this review is to provide an update on management of this disease with a focus on treatment and to give suggestions for future research. We reviewed the literature by searching the Medline database via the PubMed interface and Google Scholar for articles published between January 1999 and December 2020. The keywords used were ”acute disseminated encephalomyelitis”, “acute disseminated encephalomyelitis in children”, “ADEM and childhood”, “pediatric demyelinating disorders”, “acute disseminated encephalomyelitis and treatment”, “acute disseminated encephalomyelitis and steroid”, “ADEM and immunoglobulin”, “immunoglobulin and neurological disease”, “plasma exchange and demyelination”, “acute disseminated encephalomyelitis and plasmapheresis”, “hypothermia and acute disseminated encephalomyelitis”, and “MOG antibody”.

## 2. Insights in the Acute Disseminated Encephalomyelitis (ADEM)

### 2.1. Epidemiology

The incidence of ADEM is estimated to be 0.3 to 0.6 cases per 100,000 individuals per year, with a peak incidence during winter and spring. The geographical distribution is similar to that of MS, with a prevalence that increases with distance from the equator [3]. ADEM is usually self-limited and predominantly occurs in children and young adults. The mean age of onset is between 3.6 and 7 years, with no differences in sex [4]. 

ADEM is one of the forms of autoimmune encephalitis. It is also named post-infectious encephalomyelitis because it often occurs after an infection or more rarely after a vaccination. The infection typically comes before the onset of symptoms of approximately 2 d–4 w [5]. In most cases, it localizes in the upper respiratory tract, but in some cases, it follows an episode of gastroenteritis or, in children, an exanthematous disease. The infectious agents mainly associated are viruses, but bacteria and parasites can also be implicated (Table 1) [6]. ADEM has been associated with many vaccines, such as smallpox, measles-mumps-rubella (MMR), polio, diphtheria-pertussis-tetanus (DPT), influenza, human papillomavirus, hepatitis B, rabies, and Japanese B encephalitis, but no definitive conclusions can be made about the association of ADEM, and a specific vaccine [7]. However, the number of ADEM cases associated with vaccinations does not exceed the incidence [8].

### 2.2. Pathogenesis

**Figure 1:**
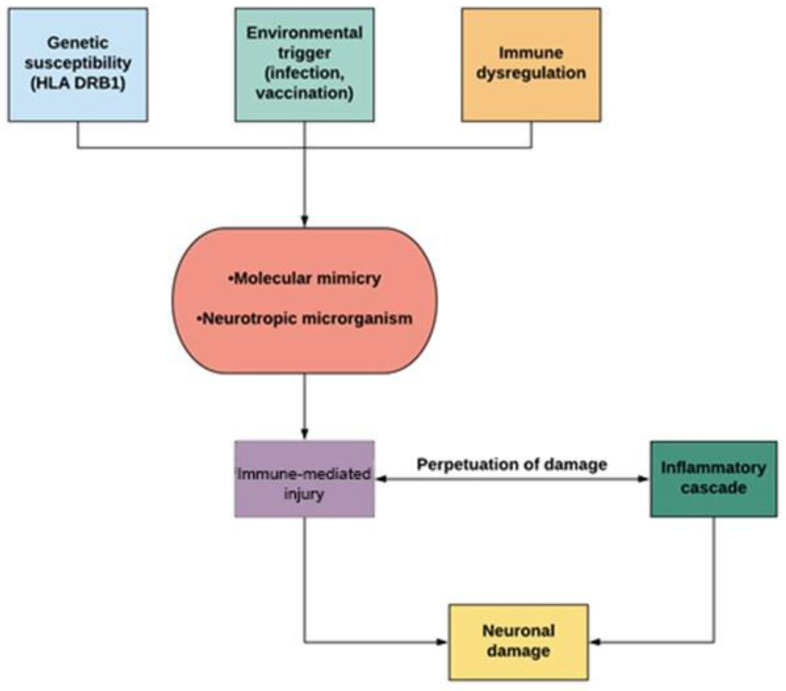
Pathogenesis of acute disseminated encephalomyelitis (ADEM).

The pathogenesis of ADEM is complex. It is thought to be the result of an autoimmune and inflammatory process of the CNS triggered by an environmental event, such as infection or vaccination occurring in genetically susceptible individuals (Figure 1). As described for MS, there is also evidence of genetic susceptibility in ADEM; indeed, patients show an increased frequency of haplotype HLA-DRB1, which seems to determine immunoreactivity to epitopes of myelin proteins. In addition, the dysregulation of the immune system can sustain this process by the breakdown of tolerance versus self-antigens [9]. 

Immune-mediated injury consists predominantly of demyelinating lesions of the CNS. ‘Molecular mimicry’ between microbial epitopes and myelin antigens, such as myelin basic protein (MBP), proteolipid protein (PLP), and myelin oligodendrocyte glycoprotein (MOG), is considered the most important mechanism causing immune-mediated injury by activating humoral and cellular immune responses. This hypothesis is supported by the presence of IgG autoantibodies and T-helper cells reactive to myelin protein in patients with ADEM [10]. Anti-MOG antibodies have been identified in both serum and cerebrospinal fluid (CSF) during the acute phase of the illness, and they have shown a progressive reduction during recovery [11]. Anti-MOG antibodies, predominantly of the IgG-1 class, are capable of triggering both cell-mediated and antibody-mediated immune responses: the antibody target has been identified in the N-terminal, immunoglobulin-like extracellular domain, in particular MOG_35–53_. The expression of the MOG protein in mature oligodendrocytes suggests its possible role in the maturation of the oligodendrocytes themselves, as well as playing a key role in maintaining the integrity of myelin, in adhesion and interaction between cells [11]. The consequent activation of an inflammatory cascade determines an increase in vascular permeability, edema, and blood–brain barrier (BBB) breakdown by the production of cytokines and chemokines, contributes to neuronal damage and causes the infiltration of immune cells in the CNS, thus sustaining the pathogenetic process [9]. This means that ADEM could be a spectrum of MOG-associated disorders. Another pathogenetic hypothesis is based on the destruction of BBB integrity due to neurotropic microorganisms that cause the release of CNS-confined autoantigens into the bloodstream [12]. 

### 2.3. Clinical Presentation

In most cases, ADEM has a monophasic course and is self-limiting, with return to neurological baseline within 3 months after the onset of symptoms. Occasionally, a subset of ADEM patients with relapsing disorders, including recurrent DEM (RDEM), multiphasic DEM (MDEM), neuromyelitis optica spectrum disorders (NMOSD), and multiple sclerosis have been reported [13]. The clinical presentation is heterogeneous. Typically, patients show prodromal symptoms such as fever, headache, malaise, nausea, and vomiting. The acute phase occurs with encephalopathy, characterized by altered behavior (e.g., irritability, confusion) and consciousness (lethargy, stupor, coma) associated with multifocal or focal neurological deficits depending on the area involved in the demyelinating process [14]. When the occipital lobe and visual cortex are involved, patients may show visual deficits, from homonymous visual field defects to cortical blindness. The involvement of associative areas of the cortex causes aphasia, alexia, agraphia, or acalculia. Pyramidal signs, such as weakness, paresis/paraplegia, hyperreflexia, spasticity, and the Babinski sign, can occur if the motor cortex is involved in the pathological process. Sensory deficits consist of agraphesthesia, astereognosis, a loss of proprioception, and altered perception of pain and temperature. Optic nerve involvement could result in optic disk edema and unilateral or bilateral optic neuritis. Brainstem involvement leads to deficits in cranial nerves III–XII (diplopia, impaired extraocular movements, dysphagia, dysarthria, nystagmus, vertigo, ataxia, hearing and taste involvement), impaired consciousness and, unfortunately, respiratory failure. Damage to the spinal cord can induce flaccid paralysis, defecation, and urinary disturbance. Seizures are not uncommon and can be focal or generalized. Other atypical symptoms include meningeal signs, dystonia, or Parkinsonism, choreiform movements, and neuropsychiatric symptoms [11]. 

Additionally, peripheral nervous system involvement has been reported in patients with signs and symptoms, including paraesthesia or anesthesia of the limbs or muscle atrophy. It has been associated with a worse prognosis and an increased risk of relapse compared to patients with only CNS involvement [15]. 

Table 2 reported the prevalence of each symptom or sign in the pediatric population. Clinical presentation is age-related and varies between children and adults, probably in view of a different immune response and consequent pattern of demyelination [16]. Pediatric ADEM is characterized by symptoms of meningoencephalitis, including encephalopathy, fever, headache, nausea and vomiting [17]. Encephalopathy is less frequent in adults, while in children it is a criterion necessary to make a diagnosis of ADEM [6]. Younger children present with altered behavior, irritability, and aggression [13]. Seizures are more common in children, especially in those younger than 5 years [18]. The involvement of the peripheral nervous system is more frequent in adult patients [19]. 

Pediatric patients with ADEM and anti-MOG antibodies usually present with encephalopathy and multifocal neurologic symptoms [2]. These patients are more likely to have complete clinical-radiological resolution after steroid therapy. Medullary involvement in subjects with anti-MOG antibodies appears to be characterized by longitudinal transverse myelitis [2].

### 2.4. Diagnosis

The diagnosis of ADEM is clinical and confirmed by neuroimaging. There are still no specific biological markers confirming the diagnosis, which is the purpose of further studies. In view of the several possible ADEM mimics, an accurate process of differential diagnosis has to be carried out, and diagnosis is made by exclusion [17]. The first priority is to rule out an acute bacterial or viral infection of the CNS, since ADEM often presents as acute meningoencephalitis. Therefore, it is necessary to perform serological and cerebrospinal fluid (CSF) analysis as soon as possible. CSF can be normal or show mild pleocytosis with lymphocytic and monocytic predominance, increased levels of protein or transient oligoclonal bands (0–29% of patients) [19]. Cultures and viral polymerase chain reaction (PCR) on CSF help to rule out infectious disease. Until an infectious etiology is excluded, patients are usually treated with broad-spectrum antibiotics and/or antiviral medications [20]. 

In diagnostic work-up, craniospinal MRI is necessary to make a diagnosis of ADEM, and is useful to rule out other causes of CNS demyelination, such as MS, neuromyelitis optica (NMO), and neuromyelitis optica spectrum disorder (NMOSD), which can overlap with ADEM in presentation [15]. ADEM usually shows areas of increased signal in T2-weighted and FLAIR images, especially involving subcortical and deep white matter as well as the spinal cord and brainstem [21]. If polysymptomatic encephalopathy occurs (i.e., movement disorder, seizures, psychosis), autoimmune encephalitis should be considered in differential diagnosis. Furthermore, very young age and family history should make clinician consider metabolic conditions, as does very symmetrical imaging [6,7]. In addition, the differential diagnosis of ADEM should include CNS malignancies, nutritional, toxic and neurometabolic disorders (especially mitochondriopathies) and autoimmune encephalitis, such as anti-NMDA receptor encephalitis, limbic encephalitis, Hashimoto encephalitis and Rasmussen encephalitis [6,7]. 

In pediatric patients, the diagnostic criteria of the International Pediatric Multiple Sclerosis Society Group (IPMSSG), published in 2007 and updated in 2013, can support the diagnosis of ADEM (Table 3). The diagnosis of encephalopathy may be especially difficult in younger pediatric patients. As a consequence, the IPMSSG criteria may underestimate the incidence of ADEM [18]. 

**Figure 2:**
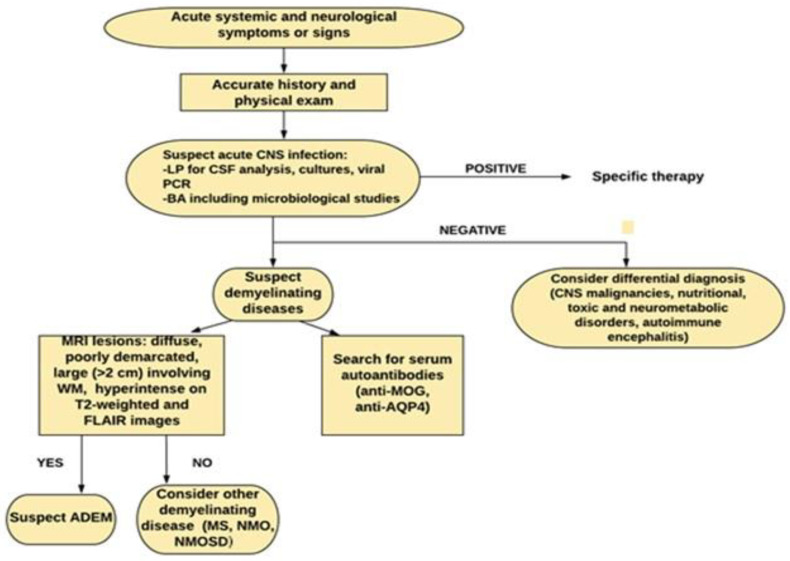
Diagnostic work-up in pediatric acute disseminated encephalomyelitis (ADEM). LP: lumbar puncture, BA: blood analysis, WM: white matter.

Any clinical symptoms and radiological findings of ADEM occurring in the following three months since the disease onset are considered part of the initial event [22]. When a second episode of ADEM occurs at least 3 months after the first episode, it is defined as multiphasic ADEM. The second episode can be characterized by new or prior neurological symptoms, signs, and MRI lesions. Three or more episodes suggest revaluing the diagnosis of ADEM and considering another chronic disorder, such as MS, MOG antibody-associated disease, or NMOSD [4]. Sometimes these disorders can converge as in ADEM-optic neuritis (ADEM-ON), which is a subtype of ADEM associated with recurrent episodes of optic neuritis [12]. In this regard, the detection of serum autoantibodies targeting CNS antigens can help to rule out a diagnosis of ADEM and to predict clinical outcome, even though their role remains to be clarified [6]. Among these, MOG antibodies have been found to be elevated in the serum of pediatric patients with ADEM during the acute phase, being correlated with younger age, male sex and with more widespread and spine longitudinally extended lesions on neuroimaging. Their presence has also been associated with seizures at presentation and post-ADEM epilepsy [14]. Patients with anti-MOG antibody positivity seem to respond to steroid therapy, showing a monophasic course and a better outcome [3], especially when conversion to seronegative status occurs [23]. The persistence of anti-MOG antibodies has been associated with multiphasic ADEM as well as with ADEM-ON [4]. However, anti-MOG antibodies are not specific and have been detected in patients with ON, transverse myelitis and NMOSD [24]. The presence of serum anti-aquaporin 4 (AQP4) IgG antibodies may help to differentiate ADEM from NMO, as they have been associated with NMO, but not with ADEM [24]. Figure 2 summarizes the diagnostic approach that we suggest to follow.

### 2.5. Outcome

Patients usually have a good outcome with a complete recovery. The outcome seems to be better in children than in young adults, especially for the disease course, recovery, and mean duration of hospitalization [20], probably due to better plasticity of the children’s brains [17]. The degree of recovery may depend in part on the underlying etiology [25]. Occasionally, rare cardiac complications have been reported [26,27]. Rubella and varicella infections have been associated with a worse outcome [19]. Mortality in ADEM is rare, except for acute hemorrhagic leukoencephalitis (AHLE), which is associated with high mortality [4]. 

## 3. Treatment

There are currently no randomized placebo-controlled trials establishing the best treatment of ADEM, which is mainly based on expert opinions and observational studies [28] and is analogous to the treatment of MS [29]. The standard is a nonspecific immunosuppressive therapy, including corticosteroids (CSs), intravenous immunoglobulins (IVIGs), and plasma exchange (PE). First-line treatment consists of high-dose intravenous CSs, followed by oral tapering. IVIGs and PE are considered second-line therapies in steroid-resistant patients, as well as in patients with contraindications to steroids. PE should be considered in the early stages of fulminant cases [6]. In addition to specific treatment, some critical patients need supportive care [30]. Rehabilitation therapy should be considered to reduce long-term sequelae [1]. 

### 3.1. Corticosteroids (CS)

Systemic corticosteroids are widely accepted as first-line therapy [31]. Approximately 94% of U.S. expert physicians specializing in CNS demyelinating diseases in children and adolescents support this approach without giving specific indications about dose and treatment duration [32]. Because of the lack of robust therapeutic trials, only class IV evidence is available (Table 4) [30].

The most commonly used therapeutic scheme for pediatric ADEM consists of intravenous methylprednisolone (IV MP) at a dosage of 10–30 mg/kg/d (maximally 1 g) or dexamethasone (DEX) 1 mg/kg/d for 3–5 days [4,20]. In a prospective study of 2002, Tenembaum et al. compared the EDSS score of 25 patients affected by ADEM with severe consciousness impairment, or optic nerve or spinal cord involvement treated with intravenous dexamethasone to that of 21 patients with similar involvement treated with intravenous methylprednisolone. The patients treated with IV MP had a lower EDSS score, as well as a better outcome [33]. 

Although these two alternative steroid formulations are both recommended, methylprednisolone is the most commonly used. Concerning oral steroid tapering, there are no clear scientific indications for either dosing or duration. Indeed, there are no randomized trials comparing the different tapering regimens. Nevertheless, some studies have shown that the risk of relapse is increased if the steroid tapering period is less than 3 w [34,35]. In most cases, oral steroid tapering starts with a dose of prednisone (PO) of 1–2 mg/kg/d and then tapers over 4–6 w. Typically, neurological improvement occurs within days after steroid treatment, and recovery is reached within a few weeks. In cases of unsatisfactory clinical improvement or early relapse, a repeat pulse of intravenous steroids may be considered. No guidelines or clinical trials support this attitude. Gupte et al. proposed a 2-w tapering course of IV MP; particularly 1-w after the initial course, a further single dose of 10 mg/kg was given, followed 1-w of another single dose of 5 mg/kg [36]. Another open question is to establish when to declare the failure of first-line treatment. Clinical features of patients were considered the most important parameters in this choice by more than 90% of the U.S. experts [32]. The natural history of ADEM is characterized by a spontaneous gradual improvement without treatment, but it may require several weeks or months [19]. In the literature, there are few data about untreated cases. In an observational study, Leake et al. compared the duration of hospitalization and hospital readmission, and found no significant differences between corticosteroid-treated and untreated patients [37]. However, the number of patients not receiving steroids was very small; thus, it is difficult to draw definitive conclusions. Experts suggest that the decision should be made on a case-by-case basis considering the clinical features of the patient and the severity of the attack [32].

### 3.2. Intravenous Immunoglobulin (IVIG) Therapy 

Based on expert consensus, IVIG therapy is recommended for the treatment of monophasic ADEM when first-line therapy with high-dose corticosteroids fails or when steroids are contraindicated. IVIG therapy may be considered for relapsing ADEM to eliminate steroid dependency [38]. IVIGs are usually administered at a dosage of 0.4 g/kg/d for 5 days or 1 g/kg/d for 2 days for a total dose of 2 g/kg given over 2–5 days [39]. 

There is a lack of an agreement about timing because there is only level IV evidence about this therapeutic option. Indeed, the use of IVIGs has been reported only in case reports and small case series [40,41,42,43,44,45] (Table 5). 

Imataka et al. described a case of an infant with ADEM not responding to a high dose of IV MP who improved after massive doses of IVIG therapy until complete recovery. This study proved the effectiveness of immunoglobulins in steroid-resistant ADEM. The authors also underlined the importance of using IVIGs in the early stages of the disease [40]. A possible predictive factor of steroid failure is the involvement of the peripheral nervous system. In these cases, IVIGs may be a therapeutic option [6]. As a second-line therapy, IVIGs could be administered alone or in association with a second course of intravenous steroids [41]. Pradhan et al. suggested that IVIGs might have a beneficial role in severe ADEM when used early [42]. They reported a case series of four patients with clinical features and MRI findings suggesting poor prognosis, among which two patients required mechanical ventilation. They were treated first with high-dose IV MP without any improvement and then were immediately treated with IVIGs undergoing a rapid recovery. However, it is not possible to rule out whether clinical improvement was due to IVIGs themselves, or to the steroids previously administered, or to a combination of both [42]. IVIGs and steroids may have a synergistic effect [43]. Therefore, there is the need for further trials to compare the efficacy of IVIGs versus steroids versus combination therapy.

Finally, some authors proposed IVIG therapy as a first-line treatment in ADEM when it is characterized by recurrent episodes [44] or when there are contraindications to steroid use [45]. In addition to the favorable effects of IVIGs, there are also risks and significant costs associated with this therapy. The rate of systemic reactions to IVIG infusion is in the range of 3% to 15%. These reactions are typically self-limited, of mild to moderate severity, and include headache, aseptic meningitis, thrombotic events, and increased risk of infection [18]. Further studies are needed to establish the safety of this treatment in children.

### 3.3. Plasma Exchange (PE)

Plasma exchange (PE) is typically used in steroid-resistant ADEM and in patients with contraindications to steroids, as an alternative to IVIGs or as a rescue therapy when response to IVIGs is not satisfactory [46]. Although PE has been shown to be superior to IVIGs in some case reports and small studies [47], the efficacy of PE compared with IVIGs is not clearly established because of the lack of randomized controlled trials comparing the two therapies [46]. For severe or life-threatening cases, plasma exchange should be considered in the early stages of the disease (class II evidence) [48]. Some authors suggested that it should be considered the first approach in fulminant ADEM [49]. Weinshenker et al. examined the efficacy of plasma exchange in the treatment of patients with severe demyelinating diseases of the CNS unresponsive to corticosteroids in a randomized controlled trial versus placebo [50]. The study revealed an improvement in the neurological status of patients treated with PE compared with those without therapy. 

PE is an established treatment in adults, but there are limited clinical data and poor literature regarding its use in pediatric patients [51]. PE therapy usually consists of 3–6 cycles, with different schedules, from every-day to every-other-day administration or over a period of 10–14 days. Because of this variability in clinical practice, randomized control trials are needed to establish the volume, frequency, duration, and timing [46]. PE is rapid-onset, and the clinical response is usually noticeable after 2–3 exchanges [52,53]. It cannot be excluded that the improvement observed during or after PE is an effect of the preceding immunosuppressant treatment because of a delayed effect of steroid/IVIG therapy, or is a part of the natural course of the disease. Some clinical and radiological characteristics could be useful to decide when to choose PE rather than other treatments. Kozioelk et al. presented a retrospective study including three cases of ADEM: two patients developed motor symptoms, and one showed recurrent ADEM with optic neuritis. After treatment with a high dose of MP and no improvement, these patients underwent PE, showing a good response [54]. Their indications for PE were an EDSS score ≥6 or severe vision loss (≤0.2) in one eye and no change in symptoms after at least 5 days of high-dose MP [54]. Khurana et al. suggested that PE might be the recommended option when there is MRI evidence of lesions in deep grey matter and the brainstem, characteristics that might be associated with a lack of response to steroid therapy and a prolonged clinical course [55]. 

The procedure for PE is safe and usually well tolerated but needs to be standardized and monitored [18,25]. Adverse reactions are rare and include moderate to severe anemia, hemodynamic instability, coagulopathy, hypocalcaemia, and allergic reactions [18]. However, it has been reported that children have more adverse events than adults (82% vs. 40%). This is probably due to the relatively increased extracorporeal volumes compared to body weight and challenges with catheter access in children compared with adults because, in children, this procedure requires vascular access via a central line [25]. Further studies are necessary to establish pediatric indications and protocols for PE in ADEM.

### 3.4. Cyclophosphamide (CYC) and Other Immunomodulatory Therapies

The use of cyclophosphamide (CYC) has been reported in adult patients with ADEM not responding to standard therapy [48]. However, little is known about its use in children. Ayed et al. described the case of a 3-year-old boy with fulminant ADEM who did not improve with high-dose intravenous methylprednisolone, immunoglobulin, or plasmapheresis. At the fifth week of illness, after one dose of intravenous CYC (750 mg/m^2^ of body surface area), he quickly improved. The authors suggested that CYC could be used in pediatric patients with fulminant ADEM after the failure of conventional treatment [10]. 

Currently, no evidence exists in favor of other immunomodulatory therapies in children with ADEM [31]. 

### 3.5. Supportive Care

Up to 25% of children with ADEM need to be admitted to the pediatric intensive care unit (PICU) due to severe encephalopathy, seizures, or diaphragm paralysis. Approximately 75% of those admitted to the PICU require mechanical ventilation [4]. Patients who develop severe brain edema could be treated with mannitol and dopamine. In single case reports, patients not responding to this medical therapy have been treated with hypothermia or decompressive craniectomy [25]. In a child with acute hemorrhagic leukoencephalitis, Yae et al. described the use of hypothermia after the development of a decerebrated posture and irregular breathing pattern, showing an improvement in both clinical and radiological features [56]. Hypothermia reduces the glial activation responsible for secondary demyelination and ensures neuroprotection. Moreover, it acts by maintaining the blood–brain barrier and inhibiting the release of excitatory amino acids or cytokines [57].

A chirurgic approach with decompressive craniectomy could be lifesaving in patients with increased intracranial pressure and signs of parenchymal compression (level IV of evidence) [48]. Granget et al. reported a pediatric case of ADEM in which, after evidence of steroid refractory intracranial pressure, emergency hemicraniectomy was performed with a favorable outcome [58]. 

### 3.6. Rehabilitation Therapy

Even though most children show excellent clinical recovery and normalization of MRI findings, neurological sequelae have been observed [59], specifically in children before 5 years of age [60]. The most common sequelae include cognitive impairment, epilepsy, visual and motor deficits (weakness, spasticity, ataxia), and impairment in speech. In these cases, it is important to start a program of early rehabilitation to ensure mobilization and the improvement of gait, muscle strength, coordination skills, and cognitive skills [48].

## 4. Conclusions

**Figure 3:**
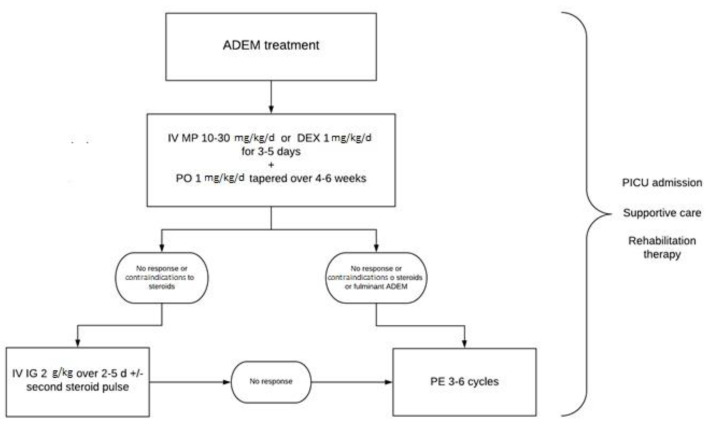
Therapeutic algorithm in pediatric acute disseminated encephalomyelitis (ADEM).

There are currently no guidelines that help clinicians manage ADEM. Indeed, there is a lack of randomized controlled trials comparing different alternative therapies and placebo-controlled trials that establish the best treatment. Figure 3 shows our adopted schedule. However, therapeutic decisions are made on a case-by-case basis. Genetic susceptibility, environmental factors, and the myelination process seem to influence the wide phenotypic variability of MOG-related syndromes.

In view of the relatively low incidence and natural course of the disease, current data about therapeutic management in pediatric patients are mainly based on long-standing case reports and small-sized studies. The results of these studies are influenced by the heterogeneity of samples in terms of age, the etiology, and severity of disease, and the time lapse between the onset of symptoms and diagnosis. Moreover, previous therapies, or the adoption of consequent treatments, make it difficult to establish the efficacy of each specific treatment. Assessed outcomes and follow-up time vary among studies. For all these reasons, it is not possible to provide a red flag list for ADEM specific treatment. High costs and ethical questions limit the availability of multicenter and placebo-controlled trials. Further studies are necessary to identify clinical, laboratory, and instrumental criteria that could be correlated with outcomes and guide clinicians in choosing when and what treatment should be given in each case. In the future, a better knowledge of the immunological basis of ADEM and the identification of the inflammatory, genetic, and biochemical factors influencing the disease course are important to discover the crucial immunological checkpoints that could be therapeutically targeted. 

Advances in understanding the association of MOG-antibodies and the serostatus of other antibodies with both monophasic and recurrent forms of ADEM must be addressed. Although in recent years the understanding about the different clinical phenotypes, diagnostic, and prognostic factors of MOG-associated disorders has significantly increased, there is still lack of evidence-based treatment protocols for acute attacks and children with a relapsing course of the disease [61]. In the acute attack, IV MP leads to a favorable outcome in the majority of patients and can be followed by tapering of oral steroids up to a maximum of three months to maintain the benefit of acute treatment by suppressing disease activity. IVIG and plasmapheresis constitute second-line therapies in case of insufficient response to IV MP. After a first relapse, maintenance treatment should be started in order to prevent further relapses and the possibility of permanent sequelae. However, many open questions on maintenance treatment remain, which need to be evaluated in further prospective studies. 

## Figures and Tables

**Table 1:** Main infectious agents involved in acute disseminated encephalomyelitis (ADEM).

Bacteria	Viruses	Parasites
*Mycoplasma pneumoniae* *Campylobacter jejuni* *Chlamydia pneumoniae* *Borrelia burgdorferi* *Legionella pneumoniae* *Leptospira* spp. *Haemophilus influenzae* type b *Streptococcus pyogenes* *Rickettsia* sp.	Cytomegalovirus Epstein–Barr virus Herpes simplex virus Human herpesvirus 6 Influenza Hepatitis A and C HIV Enterovirus Coronavirus Mumps Measles Rubella Coxsackie B Varicella zoster virus Dengue	*Toxoplasma gondii* *Plasmodium falciparum* *Cryptococcus neoformans*

**Table 2:** Prevalence of clinical features in pediatric acute disseminated encephalomyelitis (ADEM).

Symptoms/Signs of Acute Phase	Prevalence (%)
Encephalopathy	100% by definition
Fever	12–68%
Headache	6–64%
Seizures	12–50%
Cranial nerve deficits	18–39%
Speech disturbance	7–44%
Pyramidal signs	18–60%
Sensory deficits	0–9%
Cerebellar signs/ataxia	36–47%
Optic neuritis	1–15%
Urinary disturbance	6–25%

**Table 3:** International Pediatric Multiple Sclerosis Society Group (IPMSSG) diagnostic criteria for acute disseminated encephalomyelitis (ADEM).

Pediatric ADEM (All Are Required)
A first polyfocal, clinical central nervous system event with presumed inflammatory demyelinating cause; Encephalopathy that cannot be explained by fever; No new clinical and MRI findings emerging three months or more after the onset; Brain MRI is abnormal during the acute (three months) phase. Typically, on brain MRI: Diffuse, poorly demarcated, large (>1–2 cm) lesions involving predominantly the cerebral white matter; T1 hypointense lesions in the white matter are rare; Deep grey matter lesion (e.g., thalamus or basal ganglia) can be present.

MRI, magnetic resonance imaging.

**Table 4:** Main pediatric studies on corticosteroid (CS) therapy in acute disseminated encephalomyelitis (ADEM).

References	Authors/Year	Type of Study	Population	Treatment	Oral Taper	Additional Treatment	Outcome
**Acute disseminated encephalomyelitis: a long-term follow-up study of 84 pediatric patients [33]**	Tenembaum et al., *Neurology*, 2002	Prospective study	84 patients (0.4–16 years) with ADEM	80 children treated with CSs: -43 patients treated with IV DEX 1 mg/kg/day for 10 days -21 patients IV MP 30 mg/kg/day if weight ≤30 kg, 1 g/day if weight ≥30 kg for 3 to 5 days followed by PO 1 mg/kg/day for 10 days -10 patients treated with PO 2 mg/kg/day for 10 days -6 patients received oral deflazacort 3 mg/kg/day	Steroid oral tapering over 4 to 6 weeks	-29 patients: antiepileptic -58 Acyclovir -36 ICU -14 artificial ventilation	Median EDSS score of 3 (0 to 6.5) for 25 patients treated with IV DEX Median EDSS score of 1 (0 to 3) for 21 patients treated with IV MP (all patients with similar clinical involvement) (*p* = 0.029) No steroid dependency
**Acute disseminated encephalomyelitis, multiphasic disseminated encephalomyelitis and multiple sclerosis in children [34]**	Dale et al., *Brain*, 2000	Prospective study	48 children: 28 with ADEM, 7 with MDEM, 13 with MS (3–16 years)	25 patients with ADEM/MDEM treated with IV MP 30 mg/kg/day for 5 days	PO	-Antibiotics/antivirals (66%)	Comparison of mean length of steroid treatment: -relapsing MDEM group (*n* = 6) ->only 3.17 w (range 0.5–8 weeks) -non-relapsing ADEM group (*n* = 19) ->6.3 weeks (range 0.5–16 weeks)
**Acute disseminated encephalomyelitis in children: outcome and prognosis [35]**	Anlar et al., *Neuropediatrics*, 2003	Multicenter prospective study	46 patients (13 mo–15 years) with ADEM	40 patients treated with CSs at the first attack: 28 patients received IV MP 20–30 mg/kg/day for 5 days 2 patients not treated	18 patients PO 2–6 weeks	-12 patients Acyclovir -3 patients antibiotics -3 patients IVIG	High-dose MP associated with fewer complications (*p* = 0.02) Relapses in 2/8 (25%) of patients treated with high-dose MP within 7 days during first attack Relapses in 11/31 (35%) of patients who did not receive MP treatment within 7 d at the first attack. (outcome evaluated in 39 patients with follow-up >12 m) Tapering steroids over 3 w or longer associated with a lower relapse rate (difference statistically insignificant)
**Acute disseminated encephalomyelitis: a review of 18 cases in childhood [36]**	Gupte et al., J. *Paediatrics Child Health*, 2003	Retrospective study	18 children (3.5 months- 17 years) with ADEM	-8 patients: IV MP 20 mg/kg/day for 3–5 days -2 patients: IV DEX for 3–10 days -2 patients: only PO for 6 weeks -5 patients: no treatment	After IV CSs (n=10): PO 2 mg/kg/day, tapering over 4–6 weeks	-2 children with early relapses:sec ond pulse of CSs -1 patient: IVIG	Follow-up of 3 months–4 years: -good outcomes -two relapses after the cessation of steroids, complete recovery after second pulse of steroid -five children with ongoing disabilities
**Acute disseminated encephalomyelitis in childhood: epidemiologic, clinical and laboratory features [37]**	Leake et al., *Pediatric Infectious Diseases Journal*, 2004	Prospective and retrospective study	42 patients (10 months -18 years) with ADEM	-33 patients: IV MP or DEX -9 patients: no treatment	Oral CSs	- 8/33 patients treated with second-line therapy IVIGs 1 g/kg/day	No statistically significant differences between CS-treated and untreated patients regarding the duration of hospitalization (*p* = 0.43) and hospital readmission (*p* = 0.67)

**Table 5:** Main pediatric studies on intravenous immunoglobulin (IVIG) therapy in in acute disseminated encephalomyelitis (ADEM).

References	Authors/Year	Type of Study	Population	First-Line Treatment	Second-Line Treatment	Additional Treatment	Outcome
An infant with steroid-refractory cytomegalovirus-associated ADEM who responded to immunoglobulin therapy [40]	Imataka et al., European Review for Medical and Pharmacological Sciences, 2014	Case report	10-month-old boy with monophasic CMV-related ADEM	IV MP 30 mg/kg/d for 3 day-started at 9 day after onset ->no clinical improvement plus necessity of intubation	ayIVIGs at 400 mg/kg/day for 5 days started at the 15th day after onset	Continuous IV midazolam 0.3 mg/kg/hour	-Improvement in consciousness and general muscle strength since 20th d -MRI normalization 19th day-6th month -No adverse reaction -No neurological sequelae at 4 years
Acute disseminated encephalomyelitis: complication of a vaccine preventable disease [41]	Banerjee et al., BMJ Case Reports, 2018	Case report	8-year-old girl with mumps-related ADEM	IV MP 30 mg/kg/d for 5 day ->poor neurological recovery (EDSS)	aySecond course of IV MP 30 mg/kg/day for 5 days plus IVIGs 2 g/kg/day		-No residual motor deficits at 6 month of follow-up -Bladder dysfunction
Intravenous immunoglobulin therapy in acute disseminated encephalomyelitis [42]	Pradhan et al., Journal of the Neurological Sciences, 1999	Case reports	4 children (1 year–14 years) with severe ADEM (2 patients intubated upon admission)	IV MP 10–15 mg/kg/d for 3–5 days ->no improvement and severe conditions	IVIGs 400 mg/kg/d for 5 days from the next day	1 patient: oral carbamazepine	-Extubation from day 7–10 -MRI after 2 weeks: considerable resolution -Walk without support in 1–6 months
Intravenous immunoglobulin in the treatment of acute disseminated encephalomyelitis [44]	Kanaheswari et al., Medical Journal of Malaysia, 2004	Case report	3-year-old Chinese boy with recurrent episodes (3) of ADEM (most likely triggered by *S. typhi*)	IVIGs 2 g/kg/day over 5 days	None	IV Ampicillin, Cefotaxime, Acyclovir	-Response within 48 hours -No residual neurological symptoms or signs (MRI) on his first year of follow-up
Intravenous immunoglobulin therapy in acute disseminated encephalomyelitis [45]	Nishikawa et al., Pediatric Neurology, 1999	Case reports	3 children (2–5 years ) with ADEM related to gastroenteritis or mumps	IVIGs 400 mg/kg/d for 5 days	ayNone	IV antibiotic	-Improvement in consciousness in 14 hours –4 days -Complete clinical improvement in 7 days–18 days

MRI, magnetic resonance imaging.

## Data Availability

Not applicable in a review article.
